# Nomogram for Predicting the Prognoses of Patients With Pancreatic Head Cancer After Pancreaticoduodenectomy: A Population-Based Study on SEER Data

**DOI:** 10.3389/fonc.2021.766071

**Published:** 2021-11-04

**Authors:** Wei Zhang, Lin Xu, Xu Che

**Affiliations:** ^1^ Department of Hepatobiliary and Pancreatic Surgery, National Cancer Center/National Clinical Research Center for Cancer/Cancer Hospital & Shenzhen Hospital, Chinese Academy of Medical Sciences and Peking Union Medical College, Shenzhen, China; ^2^ Department of Pancreatic and Gastric Surgery, National Cancer Center/National Clinical Research Center for Cancer/Cancer Hospital, Chinese Academy of Medical Sciences and Peking Union Medical College, Beijing, China

**Keywords:** pancreatic adenocarcinoma, pancreaticoduodenectomy, prognosis, predictive model, nomogram

## Abstract

**Objective:**

In this study, we retrieved the data available in the Surveillance, Epidemiology, and End Results database to identify the prognostic factors for patients with pancreatic head cancer who had undergone pancreaticoduodenectomy and developed a prediction model for clinical reference.

**Methods:**

We screened the data between 1973 and 2015. Propensity score matching (PSM) was used to control for the confounding factors. Kaplan-Meier (log-rank test) curves were used to compare the survival rates. A nomogram was established using multifactorial Cox regression.

**Results:**

In total, 4099 patients were identified. Their median survival was 22 months, with 74.2%, 36.5%, and 26.2% survival after 1, 3, and 5 years, respectively. The median cancer-specific survival was 24.0 months, with 71.1%, 32.6%, and 21.9% survival after 1, 3, and 5 years, respectively. The results of the Cox proportional risk regression showed that age, insurance status, gender, histological type, degree of tissue differentiation, T and N stages, tumor size, extent of regional lymph node dissection, and postoperative radiotherapy or chemotherapy are independent factors affecting prognosis. PSM was used twice to eliminate any bias from the unbalanced covariates in the raw data. After PSM, the patients who had received postoperative radiotherapy were found to have a better survival prognosis and disease-specific survival prognosis than those who had not received radiotherapy [HR = 0.809, 95% CI (0.731–0.894), *P* < 0.001 and HR = 0.814, 95% CI (0.732–0.904), *P* < 0.001; respectively]. A similar result was observed for the patients who had received postoperative chemotherapy *versus* those who had not [HR = 0.703, 95% CI (0.633–0.78), *P* < 0.001 and HR = 0.736, 95% CI (0.658–0.822), *P* < 0.001, for survival and disease-specific survival prognoses, respectively]. Finally, the β coefficients of the Cox proportional risk regression were used to establish a nomogram.

**Conclusion:**

Age, insurance status, gender, histological type, degree of differentiation, T and N stages, tumor size, regional lymph node dissection, and postoperative radiotherapy or chemotherapy are factors affecting the prognosis in pancreatic head cancer after pancreaticoduodenectomy. Postoperative radiotherapy and chemotherapy can improve patient survival. These still need to be further validated in the future.

## Introduction

The new cases of pancreatic cancer in the United States in 2017 corresponded to 9% of all the cases of malignancies in the country. This cancer type is the ninth most common cancer and the fourth cause of cancer-related deaths in the United States, with an estimated 53,670 new cases and 43,090 deaths in 2017 (1). In China, there is an increasing trend in the incidence of pancreatic cancer, which has the 7th highest mortality rate among all the malignancies (2). Since there is no specific method for early detection of pancreatic head cancer and pancreatic tumors progress rapidly, patients are often lost to surgery at the time of diagnosis. Released data from the Surveillance, Epidemiology, and End Results (SEER) database show that, of the 20,470 patients diagnosed with pancreatic head cancer between 2010 and 2015, only 37.6% (7,688/20,470) had undergone surgery, with pancreaticoduodenectomy accounting for 78.5% (6,037/7,688) of all the surgical procedures. Pancreaticoduodenectomy, also known as the Whipple procedure, was first reported in 1935 by Whipple (3). This procedure is considered to be one of the most complex abdominal surgeries and characterized by high trauma and complication rates since it requires removal of numerous organs and reconstruction of the digestive tract, including the pancreas, biliary tract, and gastrointestinal tract. The median survival period of patients with pancreatic head cancer is less than two years after the surgery. In the face of such a complex procedure with extremely high postoperative complications, new methods that can effectively and accurately predict the postoperative survival rate should be identified and evaluated to establish an optimal customized treatment strategy.

The TNM staging guidelines developed by the American Joint Committee on Cancer (AJCC) are widely used in clinical practice to predict prognoses of patients with pancreatic cancer. This staging procedure is based on tumor size, extent of invasion, and lymph node and metastatic statuses. Although it is used to predict the post-surgery prognosis of patients with pancreatic cancer, its precision is often sub-optimal. There are also other potential factors affecting the prognosis, including postoperative radiotherapy or chemotherapy. Therefore, more accurate and specific prediction models than TNM staging are needed for prognostic analysis of patients with pancreatic head cancer who underwent radical pancreaticoduodenectomy.

Nomogram is a simple multivariate prediction model that incorporates multiple variables affecting prognosis to calculate the survival probability of an individual (4). Recently, disease-specific nomograms have been increasingly used for prognostic prediction in various malignancies (5–11). In addition, with the promotion and development of adjuvant therapy, treatment of pancreatic cancer has taken a multidisciplinary and comprehensive form, with surgery constituting the core. Therefore, this study aimed to develop and validate a nomogram with better applicability and higher predictive accuracy that can be used for individualized assessment of post-surgery survival of pancreatic head cancer patients undergoing pancreaticoduodenectomy.

## Materials and Methods

### Population and Inclusion Criteria

This retrospective cohort study evaluated the survival of patients with pancreatic head cancer who had undergone pancreaticoduodenectomy. The patient data were derived from the database of SEER, a cancer surveillance research program established by the National Cancer Institute in 1973 and used by the National Institutes of Health to collect the cancer statistics in the U.S. The database currently has an ongoing collection of data related to cancer incidence, prevalence, and survival rate from 18 regional registries, tracking approximately 34.6% of the U.S. population. In this study, we evaluated the prognoses of patients in the SEER database who were diagnosed with primary pancreatic head cancer between 2010 and 2015. Our exclusion criteria were i) incomplete data or missing data with important variables, including histopathological information and type of surgery, and ii) patients with secondary pancreatic cancer.

The clinical data in the SEER database include age, race, sex, primary site, histological type, degree of tissue differentiation, T and N stages, tumor size, regional lymph node dissection, radiotherapy, chemotherapy, survival time, cut-off time, survival status, and cause of death. In the SEER database, the histological grades of the tumors include grade I (well-differentiated), grade II (moderately differentiated), grade III (poorly differentiated), and grade IV (undifferentiated). Pancreatic cancer is defined using the criteria of International Classification of Diseases in Oncology, 3rd edition (ICD-O-3), code C23.9. The histological types include epithelial neoplasms (codes 8010–8049); adenomas and adenocarcinomas (codes 8140–8389); cystic, mucinous, and serous neoplasms (codes 8440–8499); ductal and lobular neoplasms (codes 8500–8549); and complex epithelial neoplasms (codes 8560–8579). We reviewed the SEER database based on the procedure record codes and the SEER Data Variable Dictionary to distinguish between the different procedure types. The item “37” in the SEER data “RX Summ-Surg Prim Site (1998+)” (NAACCR item 1290, code 37) corresponds to “Pancreatic Duodenectomy (Whipple)”. Cause-specific survival (CSS) is defined in this article as the cause of death due to pancreatic cancer. The CSS corresponds to the record “Pancreas” in the variable “COD to site rec KM.” It was calculated by the SEER database by using algorithms that extracted the cause of death from the death certificates to determine a single, disease-specific cause of death. Notably, in some cases, attribution of a single cause of death may be difficult and incorrect. For example, the cause of death may be attributed to the site of metastasis rather than the primary site.

### Statistical Analysis

Categorical variables were presented as frequencies (%), and χ2 or Fisher’s exact test was used for comparative analysis. Continuous variables were presented as mean ± SD or median [interquartile range (IQR)], and independent samples *t*-test or Mann-Whitney U-test was used for comparative analysis. Survival curves were plotted using the Kaplan-Meier method and compared using the log-rank test. The risk ratio (HR) was calculated using the Cox proportional risk model. The SEER data were extracted using SEER*Stat 8.3.5 in client-server mode. Statistical analyses were performed using SPSS Statistics 24.0 (IBM, Chicago, IL) and R software (https://www.r-project.org/) along with the optional packages of the software. Two-tailed *P*-values < 0.05 were considered statistically significant.

### Propensity Score Matching (PSM)

Another objective of this study was to assess the impact of postoperative radiotherapy or chemotherapy on survival. The uneven distribution of the patient characteristics in the SEER database population usually biases the conclusions. In this study, *via* PSM on the raw data, we controlled for covariates that would affect the treatment selection process. The covariates include identified prognostic factors and other factors or variables in the SEER data. The propensity score was considered to be the probability of a patient to receive postoperative radiotherapy or chemotherapy and estimated using a non-parsimonious logistic multiple regression model. A nearest-neighbor matching algorithm (ratio = 1: 1 without replacement) was used, and the caliper width was 0.05 SDs of the logit model used to calculate the scores.

### Multivariate Cox Regression Analysis and Nomogram Development and Validation

A multifactorial regression analysis was performed using a Cox proportional risk model. The obtained variables were included in the model to calculate the effect of these factors on the survival benefit of patients after pancreaticoduodenectomy. The β coefficients from the multifactorial Cox regression analysis were used to establish the nomogram. Calibration and receiver operating characteristic curves were used to validate the predictive performance of the model.

## Results

### Search Results and Characteristics of the Included Patients

**Figure 1:**
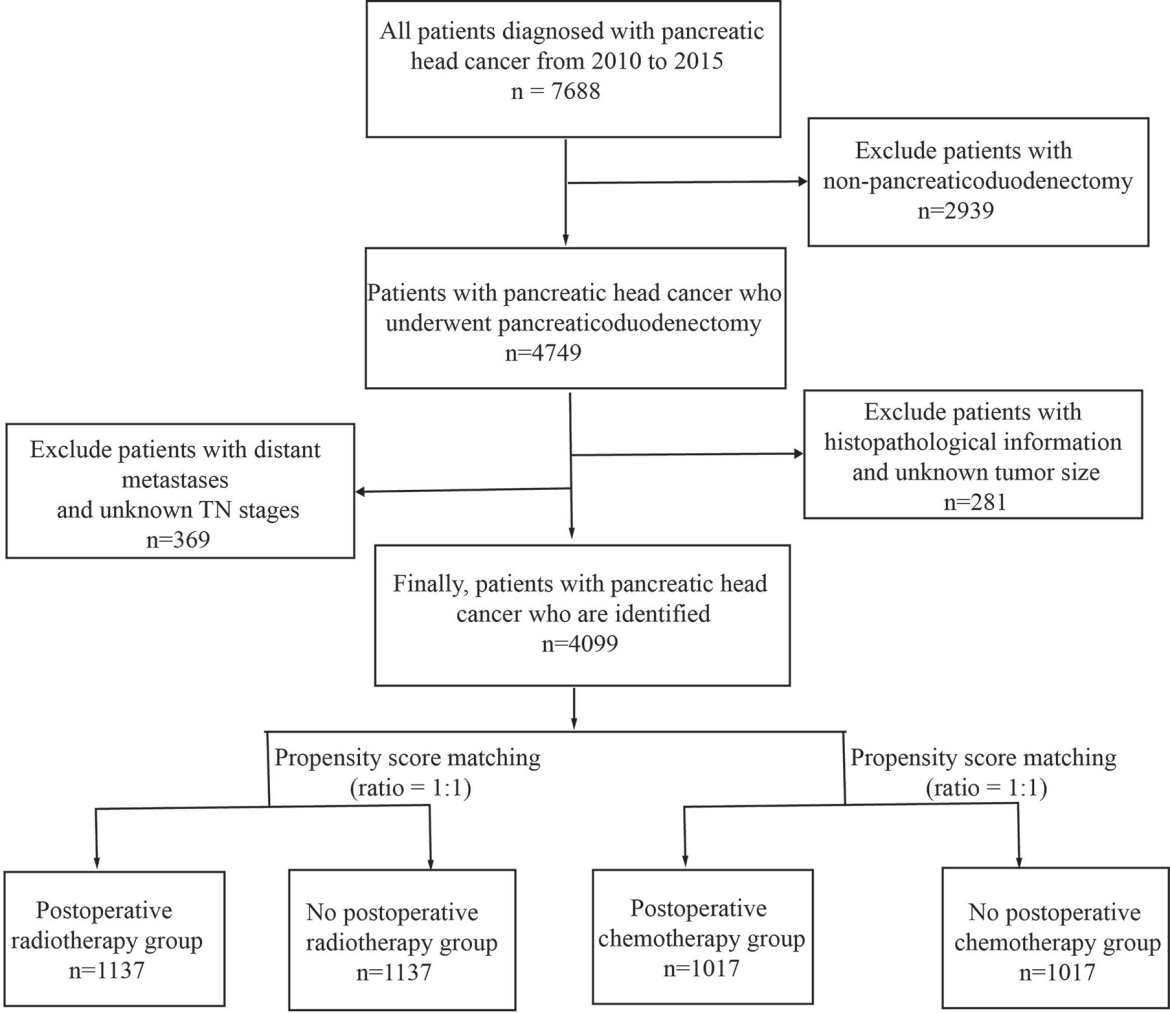
Flow chart of the study-population screening.

By applying the criteria stated above, this retrospective study initially identified that the SEER database had 7688 patients with stage M0 pancreatic head cancer who had undergone surgery between 2010 and 2015. However, we excluded 2,939 of these patients from this study because they had undergone procedures other than pancreaticoduodenectomy, such as mass enucleation and partial pancreatectomy. In addition, additional 650 patients were excluded because of unknown T or N stage, extent of lymph node dissection, pathological tissue, tissue differentiation, or tumor size. We finally identified 4099 patients. The flow chart of data acquisition and screening is shown in **Figure 1**. The demographic and clinicopathological characteristics of the patients obtained from the SEER database are shown in **Table 1**.

**Table 1:** Demographic and clinicopathological characteristics of the included population.

Variable	N = 4099, n (%) or mean (SD)
Age (year)	65.91 ± 10.74
Sex (m/f)	2116, 51.6% / 1983, 48.4%
Insurance (Medicaid/Insured/Uninsured)	409, 10% / 3603, 87.9% /87, 2.1%
Race (White/Black/Other)	3371, 82.2% /390, 9.5% /338, 8.2%
Histology classification (ICD-O-3)	42, 1.0% /2106, 51.4% /148, 3.6% /1771, 43.2% /32, 0.8%
Grade (I/II/III/IV)	632, 15.4% / 2023, 49.4%/1386, 33.8% /58, 1.4%
T stage (T1/T2/T3/T4)	248, 6.1% / 459, 11.2% / 3237, 79.0% / 155, 3.8%
N stage(N0/N1)	1324, 32.3% / 2775, 67.7%
Scope Reg LN(None/1~3/4+)	53, 1.3% / 122, 3.0% / 3924, 95.7%
Tumor size(mm)	32.71 ± 14.09
Radiotherapy (Yes /No)	2833, 69.1% / 129,3.1% / 1137, 27.7%
Chemotherapy (Yes /No)	1274, 31.1% / 2825, 68.9%

m, male; f, female; Scope Reg LN, Scope of regional lymphadenectomy ; ICD-O-3, The International Classification of Diseases for Oncology, Third Edition; SD, Standard deviation.

### Univariate and Multifactorial Cox Regression Analysis of Survival Prognosis

Univariate Cox regression analysis showed that age, gender, insurance status, histological classification, degree of tissue differentiation, T and N stages, tumor size (mm), and postoperative chemotherapy (Yes/No) or radiotherapy (Yes/No) are factors that affect the prognosis, whereas race and extent of regional lymphadenectomy are not. Multifactorial Cox regression analysis showed that age, gender, insurance status, histological type, degree of tissue differentiation, T and N stages, tumor size (mm), postoperative chemotherapy (Yes/No) or radiotherapy (Yes/No), and extent of regional lymph node dissection are independent factors affecting the prognosis, whereas race is not (**Table 2**).

**Table 2:** Univariate and multivariate cox regression analyses of the prognoses of the patients with pancreatic head cancer who had undergone pancreaticoduodenectomy.

Variable	Univariate analysis		*p*	Multivariate analysis		*p*
	HR	95% CI		HR	95% CI	
Age (year)	1.021	1.017~1.025	**<0.001**	1.017	1.013~1.021	**<0.001**
Sex						
male	Reference			Reference		
female	0.909	0.843~0.980	**0.012**	0.89	0.825~0.960	**0.003**
Insurance			**0.003**			**<0.001**
Medicaid	Reference			Reference		
Insured	0.809	0.717~0.913	**0.001**	0.735	0.650~0.832	**<0.001**
Uninsured	0.782	0.584~1.047	0.99	0.783	0.583~1.052	0.104
Race (White/Black/Other)			0.163			0.326
Histology classification (ICD-O-3)			**<0.001**			**0.001**
Epithelial neoplasms	Reference			Reference		0.296
Adenomas and adenocarcinomas	1.018	0.700~1.480	0.927	1.391	0.902~2.145	0.135
Cystic, mucinous and serous	0.731	0.475~1.126	0.156	1.184	0.732~1.916	0.49
Ductal and lobular neoplasms	1.229	0.845~1.789	0.281	1.583	1.024~2.447	**0.039**
Complex epithelial neoplasms	1.954	1.151~3.318	**0.013**	1.934	1.090~3.430	**0.024**
Grade			**<0.001**			**<0.001**
Grade I (well differentiated)	Reference			Reference		
Grade II(moderately differentiated)	1.979	1.737~2.253	**<0.001**	1.877	1.640~2.148	**<0.001**
Grade III (poorly differentiated)	2.745	2.402~3.136	**<0.001**	2.515	2.186~2.893	**<0.001**
Grade IV (undifferentiated)	2.391	1.727~3.309	**<0.001**	2.927	2.021~4.241	**<0.001**
T stage			**<0.001**			**<0.001**
T1	Reference			Reference		
T2	1.715	1.356~2.168	**<0.001**	1.296	1.016~1.653	**0.037**
T3	2.582	2.102~3.171	**<0.001**	1.675	1.345~2.087	**<0.001**
T4	3.53	2.704~4.609	**<0.001**	2.411	1.811~3.210	**<0.001**
N stage			**<0.001**			**<0.001**
N0	Reference			Reference		
N1	1.765	1.620~1.923	**<0.001**	1.669	1.524~1.829	**<0.001**
Scope Reg LN Sur			0.557			**0.002**
None	Reference			Reference		
1~3	1.098	0.735~1.639	0.649	1.042	0.695~1.562	0.842
4 or more	0.976	0.693~1.376	0.891	0.734	0.518~1.040	0.082
Tumor size(mm)	1.008	1.005~1.010	**<0.001**	1.004	1.001~1.007	**0.003**
Chemotherapy (Yes/No)						
No	Reference			Reference		
Yes	0.773	0.713~0.838	**<0.001**	0.592	0.539~0.650	**<0.001**
Radiotherapy (Yes/No)			**<0.001**			**0.011**
None	Reference			Reference		
Preoperative radiotherapy	0.8	0.643~0.995	**0.045**	1.022	0.813~1.283	0.855
Postoperative radiotherapy	0.798	0.733~0.868	**<0.001**	0.867	0.788~0.954	**0.004**

m, male; f, female; Scope Reg LN, Scope of regional lymphadenectomy; ICD-O-3, The International Classification of Diseases for Oncology, Third Edition; PSM- Propensity score matching; Bold indicates a significant difference.

### PSM and Survival Analysis of the Patients Treated With Postoperative Radiotherapy or Chemotherapy

**Figure 2:**
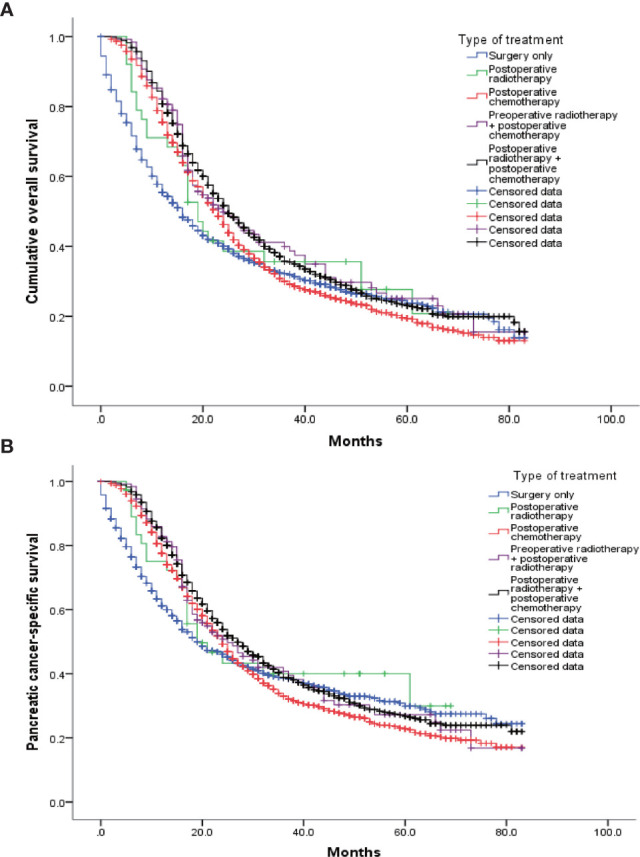
K-M survival curves for each therapy. **(A)** The log-rank test of the cumulative survival rate showed that the survival prognoses of the R+C (1597 cases), R+S+C (129 cases), and S+C+R (1099 cases) groups were significantly improved compared with that of the R group (1236 cases). In addition, there were significant differences between the S+C+R (1099 cases) and S+C (1597 cases) groups. There were no significant differences among the other groups. **(B)** The log-rank test of the pancreatic-cancer–specific survival rate showed that the R+C (1597 cases) and S+C+R (1099 cases) groups had higher rates than the R group (1236 cases). In addition, there were significant differences between the S+C+R (1099 cases) and S+C (1597 cases) groups. There were no significant differences among the other groups.

Data from 4099 patients identified from the SEER database were used for analysis. Of these patients, 1236 underwent only surgery (S group), 38 underwent surgery and postoperative radiotherapy (S+R group), 1597 underwent surgery and postoperative chemotherapy (S+C group), 129 underwent preoperative radiotherapy, surgery, and postoperative chemotherapy (R+S+C group), and 1099 underwent surgery, postoperative radiotherapy, and postoperative chemotherapy (S+R+C group). Their postoperative median survivals were 16, 19, 23, 24, and 25 months, respectively, and the pancreatic-cancer–specific median survivals were 19, 19, 24, 25, 27, and 24 months, respectively. Their all K-M survival curves are shown in **Figure 2**. The patients who received preoperative radiotherapy also received postoperative chemotherapy and were not significantly different from those who received only postoperative chemotherapy [Log Rank (Mantel-Cox) test = 0.177].

**Figure 3:**
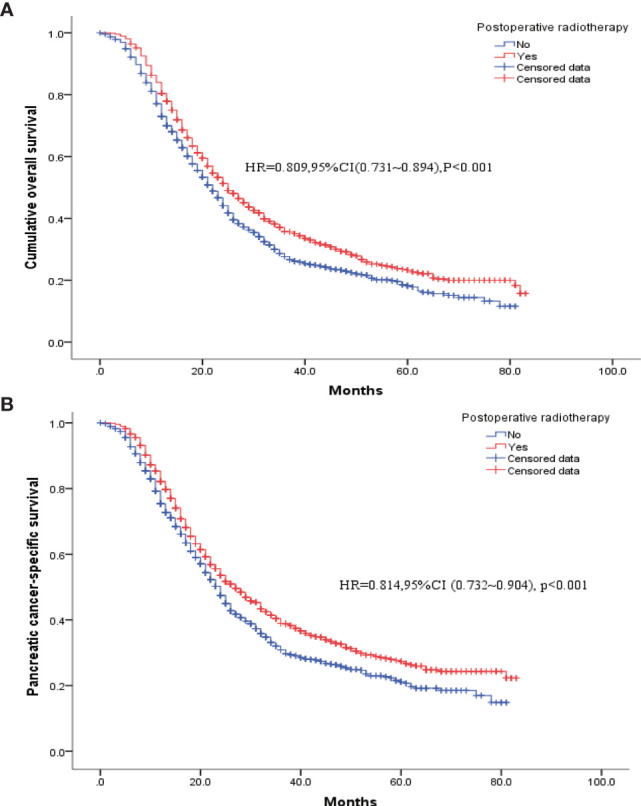
K-M survival curves for patients who had received postoperative radiotherapy *versus* those without radiotherapy after PSM. **(A)** Overall survival. **(B)** Pancreatic cancer disease-specific survival.

To eliminate the potential bias from preoperative radiotherapy, the 129 patients in the R+S+C group were removed, and the remaining patients were matched based on the propensity scores. **Table 3** shows the baseline clinicopathological characteristics of the patients divided into two groups according to whether postoperative radiotherapy was administered. The two groups had unbalanced baseline characteristics of age, tumor size, insurance status, gender, histological type, degree of tissue differentiation, T and N stages, and postoperative chemotherapy (with/without); therefore, these factors were matched as covariates for a 1: 1 propensity score to obtain two balanced groups (**Table 3**). Survival and pancreatic-cancer–specific survival rates were found to be higher in the 1137 patients who had received postoperative radiotherapy than in the 1137 patients who had not. Their median postoperative survivals were 25 and 22 months, respectively [HR = 0.809, 95% CI (0.731–0.894), *P* < 0.001], and their pancreatic-cancer–specific survivals were 24 and 27 months, respectively [HR = 0.814, 95% CI (0.732–0.904), *P* < 0.001] (**Figure 3**).

**Table 3:** Baseline characteristics of the patients in the postoperative-radiotherapy group before and after PSM.

Variable	Before PSM		*p*	After PSM		*p*
	Radiotherapy (n=1137)	No radiotherapy (n=2833)		Radiotherapy (n=1137)	No radiotherapy (n=1137)	
Age (year)	64.13±9.90	66.75±11.01	**<0.001**	64.13±9.91	64.85±10.0	0.087
Sex (m/f)	623/514	1446/1387	**0.032**	623/514	601/536	0.355
Insurance			0.107			0.562
Medicaid	93	304		93	113	
Insured	1027	2466		1027	995	
Uninsured	17	63		17	29	
Race (White/Black/Other)	942/117/78	2322/257/254	0.379	942/117/78	944/101/92	0.976
Histology classification (ICD-O-3)			0.266			0.997
Epithelial neoplasms	8	33		8	17	
Adenomas and adenocarcinomas	566	1455		566	559	
Cystic, mucinous and serous	45	100		45	36	
Ductal and lobular neoplasms	511	1220		511	513	
Complex epithelial neoplasms	7	25		7	12	
Grade			**0.04**			0.179
Grade I (well differentiated)	120	494		120	138	
Grade II (moderately differentiated)	620	1333		620	554	
Grade III (poorly differentiated)	384	962		384	424	
Grade IV (undifferentiated)	13	44		13	21	
T stage			**<0.001**			0.891
T1	35	212		35	50	
T2	99	345		99	78	
T3	955	2189		955	968	
T4	48	87		48	41	
N stage			**<0.001**			0.663
N0	279	981		279	288	
N1	858	1852		858	849	
Scope Reg LN Sur			0.526			0.144
None	10	41		10	16	
1~3	42	75		42	22	
4 or more	1085	2717		1085	1099	
Tumor size(mm)	32.44±11.63	32.62±15.03	0.679	32.43±11.63	32.40±12.98	0.944
Chemotherapy (Yes/No)			**<0.001**			1
Yes	38	1236		38	38	
No	1099	1597		1099	1099	

m, male; f, female; Scope Reg LN, Scope of regional lymphadenectomy; ICD-O-3, The International Classification of Diseases for Oncology, Third Edition; PSM, Propensity score matching; Bold indicates a significant difference.

N, no; NA, not applicable no meta-analysis conducted; PY, partial yes; Y, yes.

**Figure 4:**
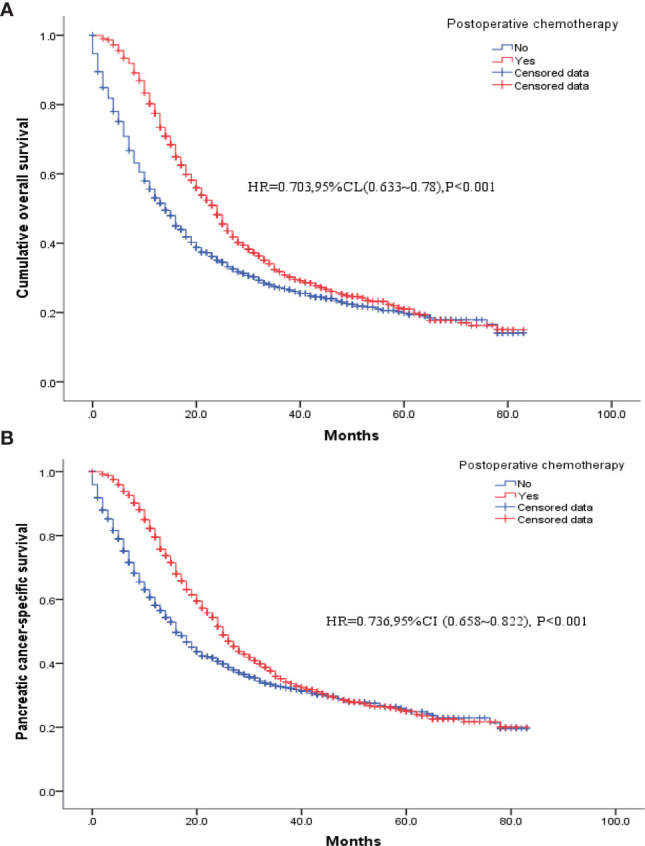
K-M survival curves for patients who had received postoperative chemotherapy *versus* patients without chemotherapy after PSM. **(A)** Overall survival. **(B)** Pancreatic cancer disease-specific survival.

**Table 4** shows the baseline clinical characteristics of the patients who had received postoperative chemotherapy *versus* those of the patients who had not. Patients who had received postoperative chemotherapy were imbalanced with those who had not in terms of age at diagnosis, tumor size, insurance status, histological type, degree of tissue differentiation, T and N stages, extent of regional lymph node clearance, and postoperative radiotherapy (Yes/No). Therefore, these factors were included as covariates in the model for 1: 1 PSM to obtain two balanced groups of patients (**Table 4**). Then, further analysis showed that the postoperative survival and pancreatic-cancer–specific survival rates of the 1017 patients who had received postoperative chemotherapy were higher than those of the 1017 patients who had not; the median survival rates were 24 and 14 months, respectively [HR = 0.703, 95% CI (0.633–0.78), *P* < 0.001], and the pancreatic-cancer–specific survival rates were 25 months and 16 months, respectively [HR = 0.736, 95% CI (0.658–0.822), *P* < 0.001] (**Figure 4**).

**Table 4:** Baseline characteristics of the patients in the postoperative-chemotherapy group before and after PSM.

Variable	Before PSM		*p*	After PSM		*p*
	Chemotherapy(n=2696)	No chemotherapy(n=1274)		Chemotherapy(n=1017)	No chemotherapy(n=1017)	
Age (year)	65.15±10.01	67.79±12.04	**<0.001**	66.90±9.97	67.53±12.08	0.193
Sex (m/f)	1413/1283	656/618	0.588	513/504	532/485	0.399
Insurance			**0.009**			0.606
Medicaid	239	158		97	114	
Insured	2408	1085		904	877	
Uninsured	49	31		16	26	
Race (White/Black/Other)	2323/275/227	1048/115/111	0.98	856/83/78	856/83/78	1
Histology classification (ICD-O-3)			**<0.001**			0.81
epithelial neoplasms	27	14		16	11	
adenomas and adenocarcinomas	1286	735		517	526	
cystic, mucinous and serous	91	54		32	39	
ductal and lobular neoplasms	1268	463		442	433	
complex epithelial neoplasms	24	8		10	8	
Grade			**<0.001**			0.52
Grade I (well differentiated)	296	318		146	160	
Grade II (moderately differentiated)	1369	584		508	499	
Grade III (poorly differentiated)	987	359		344	345	
Grade IV (undifferentiated)	44	13		19	13	
T stage			**<0.001**			0.76
T1	114	133		67	55	
T2	232	212		107	135	
T3	2259	885		809	785	
T4	91	44		34	42	
N stage			**<0.001**			0.639
N0	722	538		337	347	
N1	1974	736		680	670	
Scope Reg LN Sur			**0.025**			0.902
None	26	25		13	11	
1~3	75	42		26	27	
4 or more	2596	1207		978	979	
Tumor size(mm)	32.47±12.62	32.77±16.90	0.575	33.04±14.04	32.60±14.19	0.482
Radiotherapy (Yes/No)			**<0.001**			1
Yes	1099	38		38	38	
No	1597	1236		979	979	

m, male; f, female; Scope Reg LN, Scope of regional lymphadenectomy; ICD-O-3, The International Classification of Diseases for Oncology, Third Edition; PSM, Propensity score matching; Bold indicates a significant difference.

### Construction of a Nomogram Prediction Model

**Figure 5:**
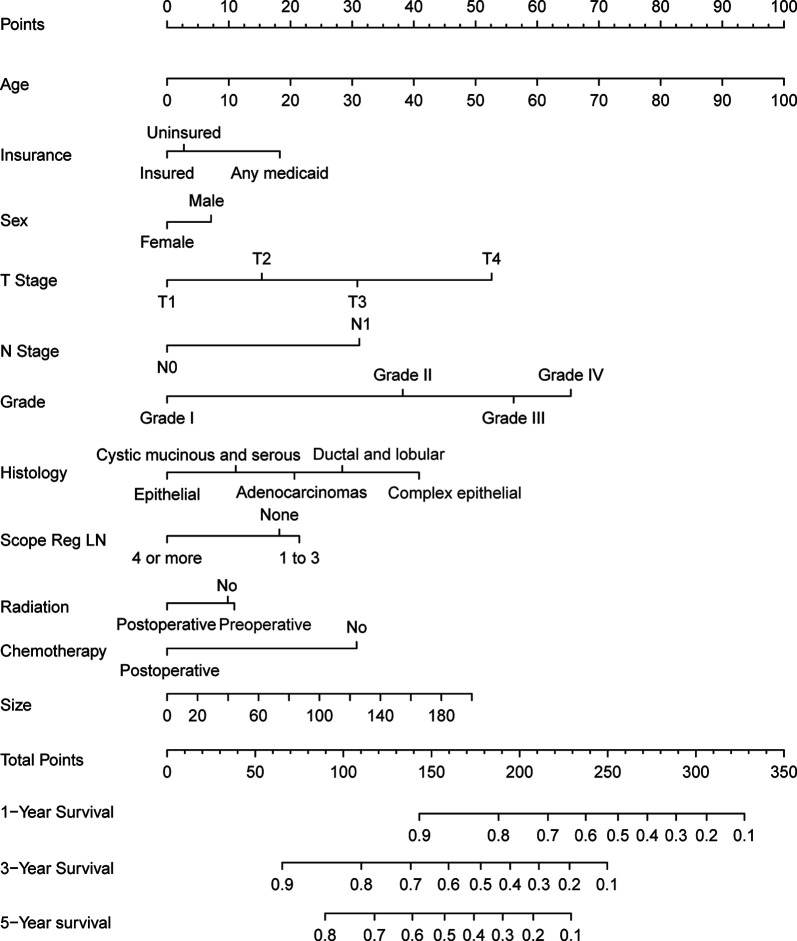
The nomogram of pancreaticoduodenectomy for pancreatic head cancer.

**Figure 6:**
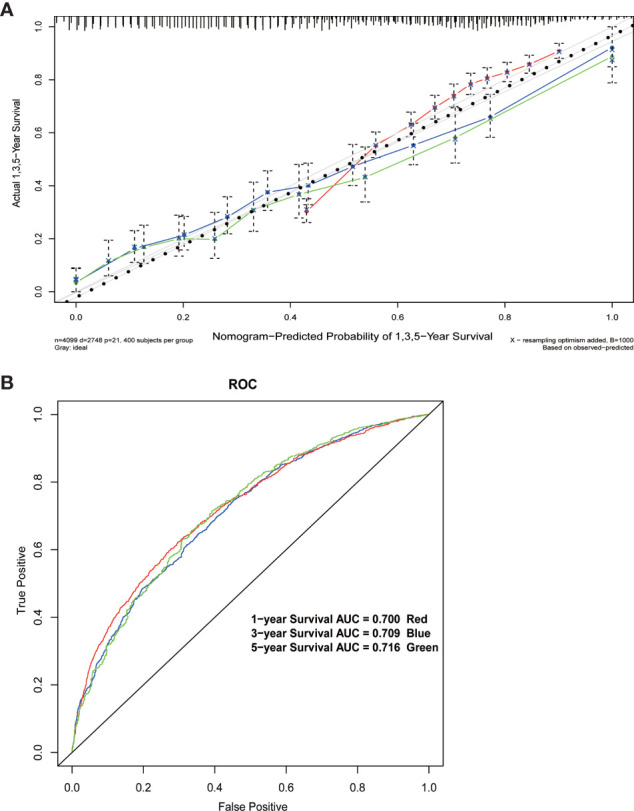
**(A)** Calibration curve of the nomogram. **(B)** 1-year, 3-year, and 5-year Receiver Operating Characteristic curves of the nomogram.

The results of the multivariate Cox regression model are shown in **Table 2**. The variables age, insurance status, gender, histology, degree of tissue differentiation, T and N stages, tumor size, regional lymph node dissection, and postoperative radiotherapy (Yes/No) or chemotherapy (Yes/No) were found to be statistically significant. The β coefficient of the model was used to establish the nomogram (**Figure 5**). The model performance was calibrated and determined using the Bootstrap internal validation method. The calibration curves showed a good identity between the predicted and actual survival outcomes (**Figure 6A**). The area under the curve (AUC) was used to evaluate the discrimination of 1-year, 3-year, and 5-year overall survival with 0.740, 0.743, and 0.743, respectively (**Figure 6B**).

## Discussion

In recent years, the incidences of carcinoma of the ampulla, distal cholangiocarcinoma, and pancreatic cancer (head, neck, and uncinate process carcinoma of pancreas) have gradually increased, and thus these cancers pose serious health risks. Pancreatoduodenectomy was developed more than 100 years ago and has evolved since then. It has been modified into many surgical procedures, including extended pancreatoduodenectomy and pylorus-preserving pancreatoduodenectomy. With the development of minimally invasive techniques and the clinical application of computers, pancreaticoduodenectomy can also be now performed using minimally invasive techniques, such as laparoscopy and robotics, and has been shown to be similar to the traditional open pancreaticoduodenectomy in terms of the outcome (12, 13). It should be noted that such minimally invasive procedures have the advantage of enhanced postoperative recovery. Although the prognosis of pancreaticoduodenectomy has improved by virtue of the technological developments and innovations in surgical techniques, this procedure is still one of the most technically complex and complicated procedures among abdominal surgical procedures. Identification of the factors affecting the prognosis of pancreatic cancer patients who have undergone pancreaticoduodenectomy will enable clinicians to customize an optimal treatment strategy.

In this study, we screened the data in the SEER database for patients diagnosed with pancreatic head cancer between 2010 and 2015 and selected the patients who had undergone pancreaticoduodenectomy. Based on the clinical variables provided by the SEER database, we found that age, insurance status, gender, histological type, degree of tissue differentiation, T and N stages, tumor size, extent of regional lymph node dissection, and postoperative radiotherapy or chemotherapy were independent factors affecting the prognosis of pancreaticoduodenectomy. We used this information to develop and validate a prognostic prediction nomogram. In addition, we also analyzed the effects of preoperative and postoperative adjuvant radiotherapies and postoperative adjuvant chemotherapy on postoperative survival. The results showed that postoperative radiotherapy or chemotherapy improves postoperative survival, whereas preoperative radiotherapy is of little benefit to patients. Because there are fewer patients receiving preoperative radiotherapy (only 129 cases) and these patients also received postoperative chemotherapy which may cause potential bias, the actual survival benefits of preoperative neoadjuvant radiotherapy still need to be further evaluated by more high-quality and better-designed studies in the future.

From the analysis results, the prognosis of patients with pancreatic head cancer after pancreaticoduodenectomy is mainly closely related to the status of the tumor itself, including T and N stages, tumor size, histology, and degree of tissue differentiation. Specifically, late T or N stage, large tumor size, or low degree of tissue differentiation is correlated with poor prognosis. In addition, patient survival outcomes also differed by insurance status, age, and gender, but there was no significant difference in postoperative survival outcome among races. Specifically, patients with Medicare had better overall survival than those without Medicare or with basic Medicare. Old age was found to be associated with poor postoperative survival. This observation may be related to more comorbidities and poorer surgical tolerance in old patients than in young patients. Interestingly, the survival prognosis of the male patients was poorer than that of the female patients, and this observation is similar to the results of previous related studies as well as previous studies on other solid tumors (14–16).

With the development and promotion of neoadjuvant and adjuvant therapies, the current treatment strategy for pancreatic malignancies is a multidisciplinary and comprehensive treatment with surgery as the core. Especially, when endoscopic ultrasound-guided fine-needle aspiration (EUS-FNA) can be used for the diagnosis of solid pancreatic lesions (17, 18), a large number of pancreatic cancers can be pathologically diagnosed before surgery. This has prompted some experts to think about whether postoperative adjuvant therapy can be treated in advance, that is, neoadjuvant therapy. But the pro-fibrotic response and cytotoxicity induced by neoadjuvant chemotherapy causes loss of healthy tissue planes and poses a challenge to any surgical procedure. However, the SEER database lacks records of preoperative chemotherapy for pancreatic cancer, preventing us from obtaining data on patients who had received neoadjuvant chemotherapy. The SEER database has only the records of pancreatic cancer patients who had received postoperative chemotherapy. In recent years, FOLFIRINOX (Fluorouracil + Leucovorin + Irinotecan + Oxaliplatin) and Nab-Paclitaxel Plus Gemcitabine have been shown to result in significant improvements in the survival of patients with pancreatic malignancies (19). In this study, approximately 69% (2825/4099) of the patients in our study had been recorded in the database to have received postoperative chemotherapy, resulting in a significantly longer survival time of 8 months (HR = 0.773, 95% CI: 0.713–0.838). Of the total 4099 patients, 1266 patients (30.9%) had received radiation therapy. The proportion receiving postoperative radiation was significantly higher in patients receiving postoperative chemotherapy than in those not receiving, and postoperative radiation therapy may have biased the effect of chemotherapy; therefore, we put unbalanced variables, including radiation therapy data, into covariates for PSM to obtain newly balanced data (1017 patients receiving postoperative chemotherapy and 1017 patients not receiving postoperative chemotherapy). The new data analysis showed that the patients who had received postoperative chemotherapy continued to have higher survival rates than those who had not received postoperative chemotherapy, with their median survivals being 24 and 14 months, respectively [HR = 0.703, 95% CI (0.633–0.78), P <0.001], and the pancreatic-cancer–specific survivals being 25 and 16 months, respectively [HR = 0.736, 95% CI (0.658–0.822), P < 0.001].

At present, this study is one of the largest studies in the real world evaluating the effects of radiation therapy in pancreatic cancer patients undergoing pancreaticoduodenectomy. In our study, 129 (3.14%) of the 4099 patients received preoperative radiotherapy and 1137 patients (27.7%) received postoperative radiotherapy. Both univariate and multifactorial Cox regression analyses found that postoperative radiotherapy was beneficial for the patients, unlike preoperative radiotherapy. Similarly, the proportion of patients receiving postoperative chemotherapy was significantly higher in patients who had received radiotherapy than in those who had not; therefore, PSM was performed to obtain balanced cohort data (1137 patients who had received postoperative radiotherapy and 1137 who did not). The results from the new analysis showed that the prognosis of the patients who had received postoperative radiotherapy was better than those who had not, and their median postoperative survivals were 25 and 22 months, respectively [HR = 0.809, 95% CI (0.731–0.894), P < 0.001], and the pancreatic-cancer–specific median survivals were 24 and 27 months, respectively [HR = 0.814, 95% CI (0.732–0.904), P < 0.001].

Radiation technology has greatly improved, and over time, radiotherapy techniques will be more precise in delivering the maximum dose to the tumor target and the minimum dose to the healthy tissue. In addition, any shift in the location of the pancreatic tumor due to respiration is taken into account *via* imaging comparisons and integration before each treatment (20). As reported by Wang et al, receiving both radiotherapy and chemotherapy was found to significantly improve the overall survival in locally advanced and metastatic pancreatic cancer within an acceptable toxicity range. A similar conclusion was obtained in the present study that patients undergoing pancreaticoduodenectomy with both radiotherapy and chemotherapy after surgery could achieve a greater benefit than those with monotherapy (**Figure 5**).

The nomogram developed in this study overcomes the shortcomings, including low specificity as well as low accuracy, of the AJCC TNM system and a previously developed nomogram (9, 21) for predicting the postoperative survival in pancreatic head cancer. When patients were predicted for survival by this nomogram, other variables, including gender, age, insurance status, tumor histological classification, degree of differentiation, extent of regional lymph node dissection, and postoperative radiotherapy or chemotherapy, together with the degree of tumor invasion, tumor size, and lymph node metastasis, were identified as prognostic risk factors. This nomogram was specially designed for patients with pancreatic head cancer who have undergone or will undergo pancreaticoduodenectomy. The results of the Bootstrap internal validation method showed that the predicted and observed values were similar for 1-, 3-, and 5-year survival rates (**Figure 6A**). From a practical point of view, the variables incorporated in the nomogram are also readily available in patients undergoing pancreaticoduodenectomy. With this simple and easily available information, clinicians can accurately predict survival and provide patients with valid information about different treatment options.

The merit of this study is that the conclusions were derived from the large population and authoritative data in the SEER database, whereby the risks of selection and publication biases were minimized. Since the SEER data had been derived from a large number of unselected patients, the conclusions of this study are universally applicable to populations, such as those in other regions of the world. In this study, after analyzing the factors affecting postoperative survival, a new prediction model was established. This prognostic prediction model may guide clinicians in the treatment of pancreatic cancer and enable them to customize effective treatment strategies.

### Limitations

Undeniably, there are several limitations in our analysis. First of all, some important laboratory data (such as CA19-9, CA125, c-reactive protein, neutrophil-to-lymphocyte ratio) and other factors that may affect the prognosis were not included in our analysis because of the lack of corresponding records in the SEER database. Although we used PSM to reduce bias, the residual confusion due to the unrecorded variables in the SEER database cannot be disregarded. Therefore, some potentially unknown influencing factors may bias the analysis results. Nevertheless, the SEER database provides sufficient sample volume to reduce possible bias, and our conclusions are still of great informative value. Second, due to the absence of neoadjuvant chemotherapy data and insufficient neoadjuvant radiotherapy data in the SEER database, we cannot accurately assess the effect of neoadjuvant radiotherapy and chemotherapy before surgery. Also, the long enrollment period of patients may also be a limitation, because the changes and improvements in surgery, chemotherapy, and radiotherapy techniques can bias the conclusions. Finally, we used the Bootstrap internal validation method to evaluate the model performance. The results showed that our prediction model has a good performance, but we do not know the accuracy of the model application since the model lacks external validation based on other cohorts.

## Conclusion

Age at diagnosis, medical insurance, gender, pathological tissue type and degree of differentiation, T and N stages of the tumor, tumor size, the intraoperative extent of lymph node dissection, and postoperative radiotherapy or chemotherapy are independent prognostic factors for pancreatic head cancer patients undergoing pancreaticoduodenectomy. Specifically, when patients are old, without health insurance, male, with pathological tissues in the order of epithelial, cystic mucinous and plasmacytic tumors, adenomas, ductal and lobular tumors, and complex epithelium, low degree of tissue differentiation, advanced T or N stage, or low regional lymphadenectomy is correlated with poor prognosis for postoperative survival. Postoperative radiotherapy or chemotherapy significantly improves patient survival. These conclusions should be verified through high-quality prospective studies in the future. The nomogram prediction model developed in this paper has a good prediction accuracy and can enable clinicians to predict the survival of a patient with pancreatic head cancer after pancreaticoduodenectomy.

## Data Availability Statement

The raw data supporting the conclusions of this article will be made available by the authors, without undue reservation.

## Author Contributions

WZ was responsible for drafting the manuscript, as well as the acquisition, analysis and interpretation of data. LX and XC contributed to the conception and design of the current study. All authors contributed to the article and approved the submitted version.

## Funding

This research supported by Sanming Project of Medicine in Shenzhen (No. SZSM 201911008 and SZSM 202011010).

## Conflict of Interest

The authors declare that the research was conducted in the absence of any commercial or financial relationships that could be construed as a potential conflict of interest.

## Publisher’s Note

All claims expressed in this article are solely those of the authors and do not necessarily represent those of their affiliated organizations, or those of the publisher, the editors and the reviewers. Any product that may be evaluated in this article, or claim that may be made by its manufacturer, is not guaranteed or endorsed by the publisher.

## References

[1] Siegel RL Miller KD Jemal A. Cancer Statistics, 2017. CA Cancer J Clin (2017) 67(1):7–30. doi: 10.3322/caac.21387 28055103

[2] He Y Liang D Li D Shan B Zheng R Zhang S. Incidence and Mortality of Laryngeal Cancer in China, 2015. Chin J Cancer Res (2020) 32(1):10–7. doi: 10.21147/j.issn.1000-9604.2020.01.02 PMC7072018 32194300

[3] Whipple AO Parsons WB Mullins CR. Treatment of Carcinoma of The Ampulla of VATER. Ann Surg (1935) 102(4):763–79. doi: 10.1097/00000658-193510000-00023 PMC1391173 17856666

[4] Iasonos A Schrag D Raj GV Panageas KS. How to Build and Interpret a Nomogram for Cancer Prognosis. J Clin Oncol (2008) 26(8):1364–70. doi: 10.1200/JCO.2007.12.9791 18323559

[5] Kong X Li J Cai Y Tian Y Chi S Tong D. A Modified TNM Staging System for Non-Metastatic Colorectal Cancer Based on Nomogram Analysis of SEER Database. BMC Cancer (2018) 18(1):50. doi: 10.1186/s12885-017-3796-1 29310604 PMC5759792

[6] Albert JM Liu DD Shen Y Pan IW Shih YC Hoffman KE. Nomogram to Predict the Benefit of Radiation for Older Patients With Breast Cancer Treated With Conservative Surgery. J Clin Oncol (2012) 30(23):2837–43. doi: 10.1200/JCO.2011.41.0076 PMC3410401 22734034

[7] Wang Y Li J Xia Y Gong R Wang K Yan Z. Prognostic Nomogram for Intrahepatic Cholangiocarcinoma After Partial Hepatectomy. J Clin Oncol (2013) 31(9):1188–95. doi: 10.1200/JCO.2012.41.5984 23358969

[8] Are C Afuh C Ravipati L Sasson A Ullrich F Smith L. Preoperative Nomogram to Predict Risk of Perioperative Mortality Following Pancreatic Resections for Malignancy. J Gastrointest Surg (2009) 13(12):2152–62. doi: 10.1007/s11605-009-1051-z 19806409

[9] Pan J Hou Y Zhang D Wu H Liu X Liu J. Efficacy and Prognostic Factors of Pancreaticoduodenectomy for Duodenal Papillary Carcinoma. J Hepatobil Pancreatic Surg (2012) 24(05):362–6. doi: 10.1007/s00330-020-06864-z

[10] Zhu S Zhu M Wang Z Chen Y. Multivariate Analysis of Pancreatic Cancer and Pancreaticoduodenectomy to Assess Prognosis. International Hepatobiliary and Pancreatic Surgery Nantong Forum and National Hepatobiliary and Pancreatic Surgery Academic Experience Exchange Conference. Chinese Medical Association; Jiangsu Medical Association (2009).

[11] Allen PJ Kuk D Castillo CF Basturk O Wolfgang CL Cameron JL. Multi-Institutional Validation Study of the American Joint Commission on Cancer (8th Edition) Changes for T and N Staging in Patients With Pancreatic Adenocarcinoma. Ann Surg (2017) 265(1):185–91. doi: 10.1097/SLA.0000000000001763 PMC5611666 27163957

[12] Zhang W Huang Z Zhang J Che X. Safety and Efficacy of Robot-Assisted *Versus* Open Pancreaticoduodenectomy: A Meta-Analysis of Multiple Worldwide Centers. Updates Surg (2021) 73(3):893–907. doi: 10.1007/s13304-020-00912-5 33159662

[13] Lyu Y Cheng Y Wang B Xu Y Du W. Minimally Invasive *Versus* Open Pancreaticoduodenectomy: An Up-To-Date Meta-Analysis of Comparative Cohort Studies. J Laparoendosc Adv Surg Tech A (2019) 29(4):449–57. doi: 10.1089/lap.2018.0460 30256164

[14] Amin S Lucas AL Frucht H. Evidence for Treatment and Survival Disparities by Age in Pancreatic Adenocarcinoma: A Population-Based Analysis. Pancreas (2013) 42(2):249–53. doi: 10.1097/MPA.0b013e31825f3af4 PMC3958965 22836862

[15] Osawa M Akuta N Suzuki F Fujiyama S Kawamura Y Sezaki H. Prognosis and Predictors of Hepatocellular Carcinoma in Elderly Patients Infected With Hepatitis B Virus. J Med Virol (2017) 89(12):2144–8. doi: 10.1002/jmv.24890 28667756

[16] Kula D Kalemba M Puch Z Polańska J Świerniak M Rusinek D. Age at Diagnosis and Gender Modify the Risk of 9q22 and 14q13 Polymorphisms for Papillary Thyroid Carcinoma. Endokrynol Pol (2017) 68(3):283–9. doi: 10.5603/EP.2017.0021 28660995

[17] Crinò SF Ammendola S Meneghetti A Bernardoni L Conti Bellocchi MC Gabbrielli A. Comparison Between EUS-Guided Fine-Needle Aspiration Cytology and EUS-Guided Fine-Needle Biopsy Histology for the Evaluation of Pancreatic Neuroendocrine Tumors. Pancreatology (2021) 21(2):443–50. doi: 10.1016/j.pan.2020.12.015 33390343

[18] Crinò SF Larghi A Bernardoni L Parisi A Frulloni L Gabbrielli A. Touch Imprint Cytology on Endoscopic Ultrasound Fine-Needle Biopsy Provides Comparable Sample Quality and Diagnostic Yield to Standard Endoscopic Ultrasound Fine-Needle Aspiration Specimens in the Evaluation of Solid Pancreatic Lesions. Cytopathology (2019) 30(2):179–86. doi: 10.1111/cyt.12662 30484917

[19] Sohal DP Mangu PB Laheru D. Metastatic Pancreatic Cancer: American Society of Clinical Oncology Clinical Practice Guideline Summary. J Oncol Pract (2017) 13(4):261–4. doi: 10.1200/JOP.2016.017368 28399388

[20] Wang Z Ren ZG Ma NY Zhao JD Zhang Z Ma XJ. Intensity Modulated Radiotherapy for Locally Advanced and Metastatic Pancreatic Cancer: A Mono-Institutional Retrospective Analysis. Radiat Oncol (2015) 10:14. doi: 10.1186/s13014-014-0312-5 25575617 PMC4296685

[21] Li HB Zhou J Zhao FQ. A Prognostic Nomogram for Disease-Specific Survival in Patients With Pancreatic Ductal Adenocarcinoma of the Head of the Pancreas Following Pancreaticoduodenectomy. Med Sci Monit (2018) 24:6313–21. doi: 10.12659/MSM.909649 PMC6144730 30198517

